# L-Carnitine Functionalization to Increase Skeletal Muscle Tropism of PLGA Nanoparticles

**DOI:** 10.3390/ijms24010294

**Published:** 2022-12-24

**Authors:** Ilaria Andreana, Manuela Malatesta, Maria Assunta Lacavalla, Federico Boschi, Paola Milla, Valeria Bincoletto, Carlo Pellicciari, Silvia Arpicco, Barbara Stella

**Affiliations:** 1 Department of Drug Science and Technology, University of Torino, I-10125 Torino, Italy; 2 Department of Neurosciences, Biomedicine and Movement Sciences, University of Verona, I-37134 Verona, Italy; 3 Department of Biology and Biotechnology “Lazzaro Spallanzani”, University of Pavia, I-27100 Pavia, Italy; 4 Department of Computer Science, University of Verona, I-37134 Verona, Italy

**Keywords:** PLGA, L-carnitine, nanoparticles, active targeting, skeletal muscle cells, fluorescence microscopy, transmission electron microscopy

## Abstract

Muscular dystrophies are a group of rare genetic pathologies, encompassing a variety of clinical phenotypes and mechanisms of disease. Several compounds have been proposed to treat compromised muscles, but it is known that pharmacokinetics and pharmacodynamics problems could occur. To solve these issues, it has been suggested that nanocarriers could be used to allow controlled and targeted drug release. Therefore, the aim of this study was to prepare actively targeted poly(lactide-*co*-glycolide) (PLGA) nanoparticles (NPs) for the treatment of muscular pathologies. By taking advantage of the high affinity for carnitine of skeletal muscle cells due to the expression of Na^+^-coupled carnitine transporter (OCTN), NPs have been actively targeted via association to an amphiphilic derivative of L-carnitine. Furthermore, pentamidine, an old drug repurposed for its positive effects on myotonic dystrophy type I, was incorporated into NPs. We obtained monodispersed targeted NPs, with a mean diameter of about 100 nm and a negative zeta potential. To assess the targeting ability of the NPs, cell uptake studies were performed on C2C12 myoblasts and myotubes using confocal and transmission electron microscopy. The results showed an increased uptake of carnitine-functionalized NPs compared to nontargeted carriers in myotubes, which was probably due to the interaction with OCTN receptors occurring in large amounts in these differentiated muscle cells.

## 1. Introduction

For years, the enhanced permeation and retention (EPR) effect was the chosen strategy for passive targeting, being largely studied for nanocarrier accumulation in a tumor environment [1,2]. However, models used to study the EPR effect are often not sufficiently accurate to describe the nanocarrier behavior in the human body [3]. The active targeting approach is based on the identification of precise disease biomarkers, to which specific ligands on the nanocarrier surface are directed to promote accumulation into organs or tissues with consequent improved therapeutic effects. Furthermore, the high specificity of the ligands (e.g., antibodies, peptides) can facilitate nanocarrier internalization into cells with great affinity and precision [4].

Muscular dystrophies (MDs) are a group of chronic inherited genetic diseases which affect muscles, especially at the skeletal level. The most common MDs are represented by Duchenne muscular dystrophy and myotonic dystrophies, caused by different and specific gene mutations [5,6]. Currently, no therapies are available to counteract the pathogenic causes of MDs, and conventional treatments are based on glucocorticoids to mitigate symptoms [7]. Over the last years, various approaches have been proposed for the treatment of localized mutations. For instance, gene therapy is considered the most recent strategy to target the pathogenic causes of MDs [8,9]. However, drawbacks related to the instability of the genetic materials or the unreached global distribution to all compromised tissues limit the therapeutical applicability of this strategy. Within this framework, drug delivery systems have been proposed for the treatment of MDs. However, in vivo delivery is challenging due to the presence of biological barriers: the complex architecture of skeletal muscle and the surrounding extracellular matrix (ECM) made of fibrous proteins are the main hurdles for drug delivery to muscle cells [10,11]. To restrict interactions with ECM, the intravenous administration of nanocarriers appeared to be a potential strategy to reach skeletal muscle fibers through their dense blood capillary network. However, the treatment of MDs requires long-term administration, which makes the biocompatibility and biodegradability of nanosystems important requirements.

In this context, surface-engineered nanosystems have been designed to actively promote muscle cell targeting thanks to peptides anchored on the nanocarrier surface [12,13]. In our work, we identified the Na^+^-coupled organic cation/carnitine transporter (OCTN) as a potential target for drug delivery at the muscular level [14,15]. OCTN receptors constitute a subfamily of the solute carrier SLC22 family, whose differences in amino acid composition define the two main receptor classes, namely OCTN1 and OCTN2. Moreover, each member of SLC22 has differences in substrate affinity. In particular, OCTN2 has a high affinity for L-carnitine, which is a small zwitterion molecule endogenously synthetized by liver, kidney and brain [16].

L-Carnitine-associated polymer nanoparticles (NPs) have been shown to be effective in increasing drug delivery by oral administration: the insertion of an L-carnitine derivative, namely stearoyl-L-carnitine (SC), into the polymer matrix enhanced cellular uptake and intestinal absorption of drug molecules by OCTN2-mediated transport [17,18]. Furthermore, SC-conjugated PLGA NPs have been described as novel potential tools for colon cancer cell-targeted drug delivery. Indeed, cancer cells express high levels of OCTN2 and ATB^0,+^ receptors, both having high affinity for L-carnitine [19]. The same approach can be exploited to target the respiratory epithelium of human trachea and bronchi, since the identification of OCTN transporters can play a significant role in the delivery of therapeutic molecules by a nanomedicine-based approach [20,21].

**Figure 1:**
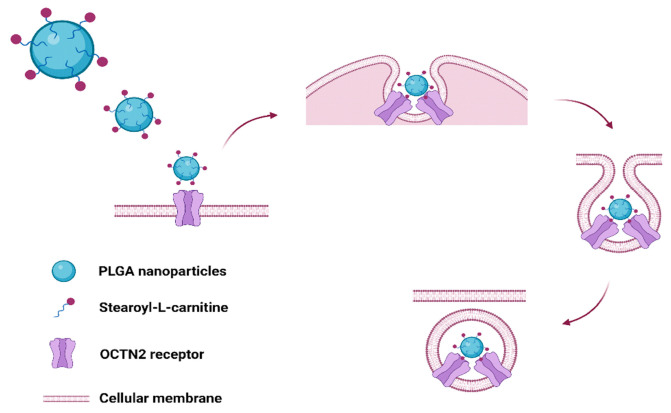
Cellular uptake of SC-PLGA nanoparticles: functionalized nanoparticles bind to OCTN2 receptor on the cell membrane, forming a complex that is then internalized into the cell (created with BioRender.com).

On these bases, in the present work we propose the formulation of drug-loaded SC-associated PLGA NPs to target the OCTN2 receptor expressed on skeletal muscle cells (Figure 1).

We tested different percentages of SC to efficiently functionalize the NPs, which were then characterized. In vitro tests were carried out in an established non-tumor muscle cell line, with cells able to proliferate as myoblasts (mimicking activated satellite cells of skeletal muscle) and terminally differentiate into myotubes (mimicking the mature myofiber) [22]. Cytotoxicity in the presence of targeted NPs was assessed, and cellular uptake was analyzed by confocal and transmission electron microscopy (TEM).

The recent understanding of the pathogenic mechanism of MDs has opened new possibilities for innovative strategies. As an example, a current approach for the treatment of myotonic dystrophy type I consists in drug repurposing, i.e., the identification of new therapeutic applications of existing drugs [23]. Within the frame of the repurposing approach, pentamidine (PTM), an aromatic diamine approved as an antiparasitic drug, has been investigated as an experimental MD treatment. Indeed, myotonic dystrophy type I results in the dystrophia myotonica protein kinase (DMPK) gene mutation, which leads to the amplification of the (CTG)_n_ triplets. Furthermore, the transcription of expanded (CUG)_n_ forms aggregates of hairpin structures in the nucleus, namely nuclear foci, responsible for the sequestration of the splicing factor muscle-blind-like (MBNL) protein family and the consequent myotonia and muscle weakness [24,25]. Recent in vitro and in vivo studies demonstrated that PTM associates with the expanded triplets, preventing MBNL1 sequestration, decreasing the formation of nuclear foci and reducing myotonia in experimental models [26,27]. However, its substantial toxicity at the potentially effective dose in vivo and its relevant side effects prevent its clinical applicability [28]. For these reasons and to improve drug efficacy, PTM has been encapsulated into targeted NPs for its proposed activity in the treatment of myotonic dystrophy type I [8,29,30,31].

## 2. Results

### 2.1. Preparation and Characterization of SC-Nanoparticles

Empty and drug-loaded SC-associated (5% and 10% *w*/*w*) PLGA NPs were prepared in a single step by nanoprecipitation [32], adding polyvinyl alcohol (PVA) in the aqueous phase as a stabilizer of the composite formulation. As reported in Table 1, all the NP samples showed a mean diameter of about 100 nm; in particular, the mean size value of empty NPs was lower than that of drug-loaded samples. Indeed, the incorporation of the free base form of PTM (PTM-B) increased the mean size until it grew to about 130 nm and the polydispersity index (PDI) value was greater than 0.2. Concerning the zeta potential, SC-associated and PTM-B-loaded NPs showed a less negative value than that of blank nanocarriers, thanks to the presence of positive charges on both SC and PTM-B.

**Figure 2:**
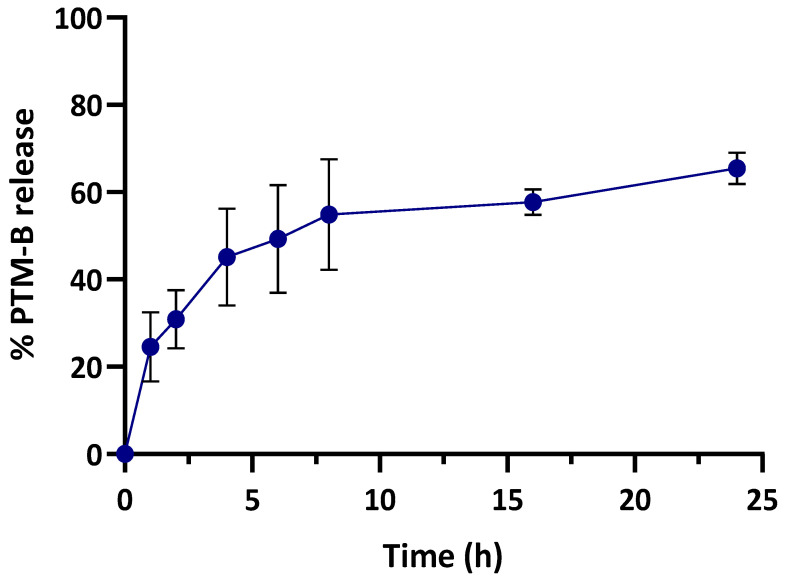
PTM-B release from 5% SC-nanoparticles as a function of time after incubation in PBS pH 7.4 at 37 °C.

The physical stability of the NP suspensions in the storage conditions was monitored for four weeks; in this period, no precipitation and/or aggregation occurred for 5% and 10% empty SC-NPs. On the contrary, for PTM-B-loaded nanosystems, the mean particle size, PDI and zeta potential values were stable only for 5% SC-NPs. On these bases, we selected the lower SC concentration for further characterization. In particular, 5% SC-PTM-B-PLGA NPs showed an encapsulation efficiency (EE) (calculated as the ratio between the amount of entrapped drug and the initial amount used in the preparation of nanocarriers × 100) of 65% and a drug loading (DL) (calculated as the ratio between the amount of entrapped PTM-B and the total nanocarrier weight × 100) of 1.9%. This EE was lower than that of untargeted NPs, which was about 90% [33]. HPLC analysis showed that 45% of the initially added SC was associated with 5% SC-PTM-B-PLGA NPs (corresponding to a final SC concentration of 22.5 µg/mL). To evaluate the PTM-B release from 5% SC-NPs, the suspension was incubated for 24 h at 37 °C in phosphate-buffered saline (PBS) pH 7.4. In these conditions, the drug is stably associated to the polymer matrix thanks to electrostatic interactions, as previously reported [33]: indeed, after 24 h, only 60% of PTM-B is released (Figure 2).

### 2.2. Cytotoxicity Assay

**Figure 3:**
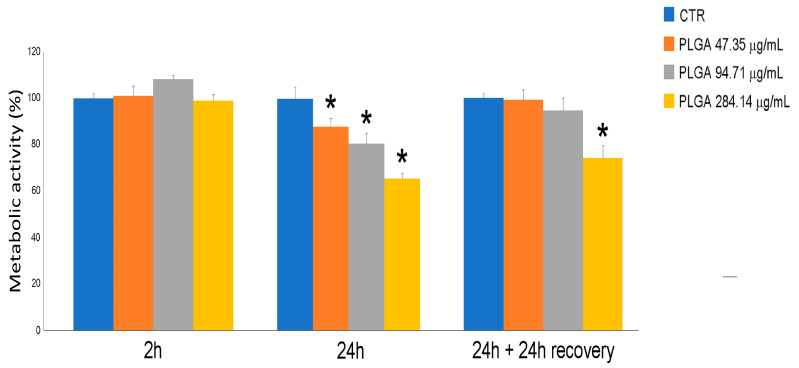
C2C12 myoblast metabolic activity, evaluated by MTT assay after 2 h, 24 h, 24 h + 24 h recovery treatment, in presence of 5% SC-nanoparticles. Data are given as mean values ± SEM, representative of three independent experiments. * *p* < 0.03 vs. control (CTR) sample.

C2C12 myoblasts were cultured and incubated for 2 h, 24 h and 24 h + 24 h of recovery in a culture medium without NPs, with increasing concentrations of 5% SC-NPs used to evaluate cell metabolic activity. As shown in Figure 3, metabolic activity was similar in control samples and in samples exposed to any nanocarrier concentration after 2 h of incubation. Conversely, metabolic activity decreased significantly after 24 h incubation in a dose-dependent manner (from 86% for the lowest concentration to 62% for the higher concentration), indicating a cytotoxic effect. After 24 h incubation with 5% SC-NPs followed by 24 h of recovery, metabolic activity showed similar values in control samples and in samples exposed to 5% SC-NPs. The exception was the highest concentration, which induced a significant decrease in 24%.

### 2.3. Nanoparticle Distribution in Myoblasts and Myotubes

**Figure 4:**
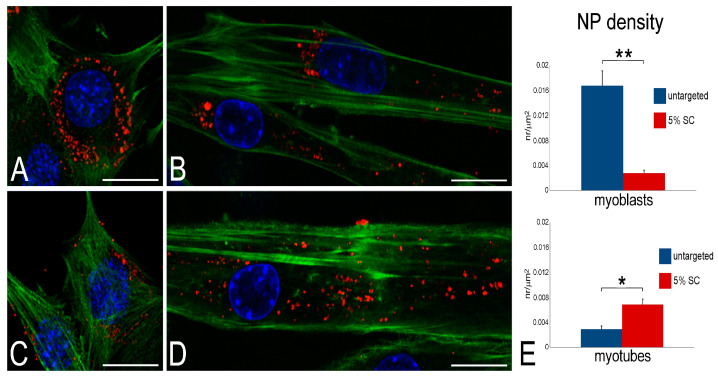
(**A**) CFM micrographs of myoblasts (**A**,**C**) and myotubes (**B**,**D**) incubated with untargeted nanoparticles (**A**,**B**) and 5% SC-associated nanoparticles (**C**,**D**). Nanoparticles are loaded with Nile Red (red), cells are stained with phalloidin (green) and nuclei with Hoechst 33,342 (blue). Bars, 20 µm. (**E**) Quantitative evaluation of internalized nanoparticles; data are given as mean values ± SEM. ** *p* < 0.001; * *p* < 0.01.

To compare the cell internalization of 5% SC-associated or untargeted NPs, C2C12 myoblasts and myotubes were incubated for 2 h and 24 h with fluorescent (Nile Red-loaded) NPs. Confocal microscopy (CFM) analysis showed that both NP types were already internalized after 2 h (not shown) and accumulated in the cytoplasm after 24 h without entering the cell nucleus (Figure 4A–D).

Morphometric analysis, performed to quantify the internalized NPs after 24 h incubation, demonstrated that untargeted NPs accumulated in myoblasts in significantly higher amounts than 5% SC-NPs. Conversely, in myotubes a higher number of 5% SC-NPs was internalized compared to the untargeted ones (Figure 4E).

**Figure 5:**
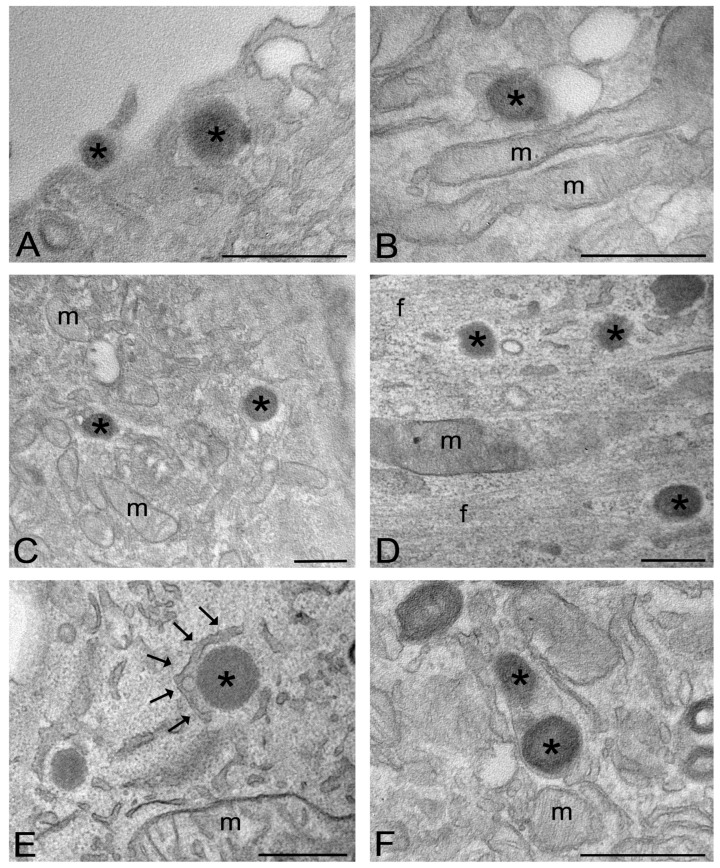
TEM micrographs of myoblasts (**A**–**C**,**F**) and myotubes (**D**,**E**) after 2 h (**A**,**B**) and 24 h (**C**–**F**) incubation with 5% SC-nanoparticles, respectively. (**A**) Two nanoparticles (asterisks) are entering the cell by endocytosis. (**B**) A nanoparticle (asterisk) is escaping an endosome. (**C**,**D**) Nanoparticles (asterisks) occur free in the cytosol. (**E**) A nanoparticle, occurring free in the cytosol (asterisk), is enclosed by double membranes (arrows) undergoing autophagocytosis. (**F**) Two nanoparticles (asterisks) inside a secondary lysosome. m, mitochondria; f, myofibril bundles. Bars, 200 nm.

TEM analysis confirmed the uptake of 5% SC-NPs within myoblasts and myotubes (Figure 5) after 2 h and 24 h incubation, allowing us to shed light on the NP interaction with the cellular components. At TEM, NPs showed a regular roundish shape and a moderate electron density, conditions which allowed their unequivocal visualization.

The intracellular fate of 5% SC-NPs was the same in myoblasts and myotubes. After 2 h of incubation, 5% SC-NPs were found to enter the cells by endocytosis (Figure 4A). Once in the cytoplasm, they underwent endosomal escape (Figure 4B). After 24 h incubation, most NPs were free in the cytosol (Figure 4C,D). However, some NPs were found to be partially surrounded by double membranes as a typical sign of the autophagic process (Figure 4E), while secondary lysosomes containing NP remnants accumulated in the cytoplasm (Figure 4F). NPs were never found inside the nucleus. Only several cells showed signs of stress, such as vacuolization or mitochondrial swelling, at 24 h incubation (not shown).

### 2.4. Immunofluorescence Detection of OCTN2 Receptor

**Figure 6:**
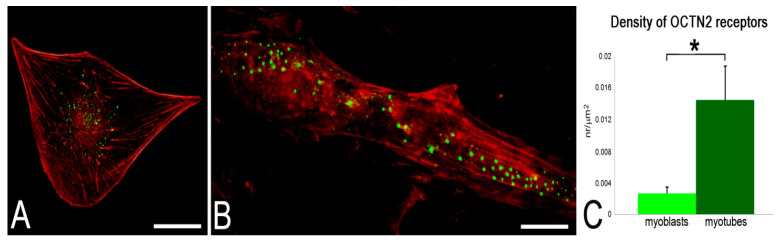
CFM micrographs of a myoblast (**A**) and a myotube (**B**), immunolabelled for OCTN2 (green); counterstaining with phalloidin (red). Bars, 20 µm. Note the higher density of receptors in the myotube (**C**); data are given as mean values ± SEM. * *p*< 0.001.

To detect the presence of OCTN2 receptors in myoblasts and myotubes in order to gain further insight into the possible role of the transporter, the cells were labeled with the anti-OCTN2 antibody and evaluated by immunofluorescence. The presence of OCTN2 receptors was observed in both myoblasts and myotubes, but in myotubes the number of receptors was significantly higher (Figure 6).

## 3. Discussion

In this work, L-carnitine was exploited as an active targeting agent to increase the muscle tropism of PLGA NPs and improve the therapeutical treatment of MDs. L-carnitine is a specific substrate for a plasma membrane transporter, namely OCTN2, a Na^+^-coupled transporter that is characterized by high binding affinity for L-carnitine (K_m_~10 µM) [34]. PLGA NPs themselves are poorly taken up by differentiated muscle cells [35,36], but chemical modification of the surface of these NPs with a specific targeting molecule would potentially enhance the efficiency of the uptake process and cell selectivity. The rationale of this study is that differentiated muscle cells preferentially express OCTN2 receptors, which recognize L-carnitine as substrate.

To promote the L-carnitine association to PLGA NPs, we have chosen a commercial derivative, i.e., SC, in which the hydroxyl group of L-carnitine was conjugated to a stearoyl moiety. In this way, during NP formation, the SC long fatty chain anchors to the PLGA matrix by hydrophobic interactions and exposes hydrophilic L-carnitine on the NP surface (Figure 1) [19]. According to this approach, the covalent linkage between PLGA and L-carnitine can be avoided. A similar strategy has been used to prepare L-carnitine-functionalized NPs by solvent extraction/evaporation to increase drug oral delivery by carnitine receptor-mediated uptake [18].

In this work, PTM, a drug repurposed for potential applications in MD treatment [26], was encapsulated in muscle-targeted NPs. We used the free base form of PTM to increase its hydrophobicity and, thus, the incorporation into the lipophilic inner core of the NPs. At the same time, the association of PTM-B to PLGA was promoted by the electrostatic interactions between the positively charged PTM-B amidinic groups and the negatively charged PLGA carboxyl functions, as previously reported [33].

All the NPs were prepared by nanoprecipitation, adding PVA as a surfactant to stabilize the nanosystems. In previous studies, different concentrations (from 0.1 to 1% *w*/*v*) of PVA were assessed to obtain stable NPs, limiting, at the same time, PVA toxicity in vitro. Finally, a nontoxic 0.2% PVA concentration was used [37].

NPs were formulated at 5% and 10% SC to investigate the effect of the ligand percentage on the NP characteristics. As reported in Table 1, the NP mean diameter tends to diminish with the increase in SC percentage during NP formation, probably as a consequence of the amphiphilic character of SC. On the contrary, the incorporation of PTM-B into functionalized NPs causes mean size and PDI to increase: this effect may be due to the more complex composition of the systems and the localization of PTM-B inside the NP matrix. Moreover, the zeta potential of targeted NPs was higher than that of untargeted ones, suggesting that SC is located on the NP surface. When PTM-B is added, the zeta potential value becomes even less negative due to the presence of the positively charged groups of the drug. Since the stability of the drug-loaded nanosystems was guaranteed only at 5% of SC, we evaluated the EE only for this sample, showing that the insertion of SC in the polymer matrix reduced the incorporation of PTM-B from 90% of untargeted NPs to 65%. This result is probably caused by the interaction of the stearoyl chain of SC with the polymer matrix and the competition with PTM-B incorporation. However, the drug release profile demonstrated a stable association of PTM-B with the polymer matrix thanks to the electrostatic interactions, as previously reported [33].

When administered to myoblasts in vitro, 5% SC-NPs were proved to exert a slight toxicity in a dose-dependent manner. Since PLGA is a biodegradable and biocompatible polymer without toxic effects in vitro and in vivo [38], the increased cytotoxicity may be related to the presence of SC. Indeed, some studies demonstrated the potential toxicity of acylcarnitine derivatives. Accordingly, a high level of acylcarnitine derivatives is an indicator of compromised lipid metabolism [39]. Furthermore, in cardiomyocytes, acylcarnitine has been shown to disrupt the sarcolemmal integrity and electrophysiologic functions, possibly leading to the alteration of myocardial activity [40]. Taking into account that lipid metabolism occurs in mitochondria where the dehydrogenases responsible for tetrazolium salt MTT reduction are located, and that L-carnitine is a key element for mitochondrial homeostasis, we can assume that SC would interfere with mitochondrial activities, thus reducing myoblast metabolism. These effects seem to be transitory and reversible, as suggested by the resumption of cell metabolism after a 24 h-recovery.

The observations, made at CFM and especially at TEM, demonstrated that a low number of cells, treated with 5% SC-NPs at the PLGA concentration of 94.71 µg/mL, showed stress signs after 24 h incubation according to the MTT assay results. In this study, we selected this concentration for microscopy analyses due to technical reasons. In fact, the sample sections for TEM are very thin (70–90 nm), allowing the observation of a limited cell volume. Therefore, to be sure to have performed an adequate sampling of internalized NPs at the ultrastructural level, it was advisable to use a relatively high NP concentration while preserving the viability of most of the cell population. Of note, in view of future studies with targeted drug-loaded NPs, these PLGA concentrations correspond to a nontoxic amount of entrapped PTM-B. Under these experimental conditions, it was therefore possible to monitor the uptake and intracellular fate of 5% SC-NPs. Both CFM and TEM demonstrated that 5% SC-NPs rapidly enter myoblasts and myotubes and accumulate in the cytoplasm but never penetrate the cell nucleus, thus avoiding the unpredictable side-effects due to possible interactions between NPs, nucleic acids and nuclear factors confined inside the nuclear envelope. This finding should be therefore considered as a biocompatibility feature of 5% SC-NPs.

The experimental evidence at TEM indicates that 5% SC-NPs enter myoblasts and myotubes by the classical endocytic process [18,41]. Once inside the cell, 5% SC-NPs rapidly escape the endosomes and occur free in the cytosol, similarly to what was previously reported for untargeted PLGA NPs in muscle cells [36] as well as for other polymeric NPs [42,43]. However, the free NPs re-enter the endolytic pathway due to autophagic process, thus undergoing enzymatic degradation, as demonstrated by the large number of secondary lysosomes containing NP remnants at the longer incubation time. The degradation of 5% SC-NPs through a physiological pathway further supports the biocompatibility of this nanosystem.

Although 5% SC-NPs proved to enter both myoblasts and myotubes, morphometric analysis, which was performed at CFM to quantify the NP amounts, revealed that these nanocarriers were better internalized in myotubes than in myoblasts. In detail, untargeted NPs were internalized in large amounts in myoblasts and in lower amounts in myotubes, whereas L-carnitine-functionalized NPs were internalized in higher amounts in myotubes than in myoblasts. The more efficient uptake of untargeted PLGA NPs in myoblasts with respect to myotubes was already reported in both murine C2C12 cells and human primary muscle cell cultures [35,36], being ascribed to multiple factors affecting nanocarrier internalization [44]. These include: (i) the higher metabolic rate of cycling cells in comparison to terminally differentiated ones [45]; (ii) the differential expression in myoblasts and myotubes of many proteins involved in e.g., adhesion, transmembrane transport, cytoskeleton dynamics [46,47,48], and in plasmalemma lipid composition [49]; (iii) the remarkable difference in cell size [50]; (iv) a different protein corona [51] due to the different composition of the culture media. It is likely that the enhanced uptake of targeted NPs in myotubes is due to the higher amount of OCTN2 receptors in comparison to myoblasts. In fact, OCTN2 is considered as the most important plasma membrane carnitine transporter [16,52]. Both myoblasts and myotubes are known to express OCTN2 receptors [53], but the functional activity induced by the differentiation process, such as spontaneous contractile activity, may induce in myotubes the translocation of OCTN2 receptors to the plasma membrane, similarly to what was observed in skeletal muscle in vivo [54]. Accordingly, in vitro studies on human primary cell cultures suggested that the carnitine transport system gradually develops during myogenesis before being fully expressed in the adult tissue [55].

## 4. Materials and Methods

### 4.1. Materials

PLGA 75:25 (Resomer^®^ RG 752 H, Mw = 4–15 kDa) (analytical grade), SC, PVA (Mw = 31–50 kDa, 98–99% hydrolyzed), PTM isethionate (PTM-I), dimethyl sulfoxide (DMSO), phosphoric acid, sodium hydroxide and sodium 1-heptanesulfonate were purchased from Merck (Milan, Italy). All the solvents used were of analytical grade or HPLC grade and were purchased from Carlo Erba Reagenti (Milan, Italy). Ultrapure water used for the buffers was obtained from a Milli-Q^®^ Plus Purification System (Merck Millipore, Vimodrone Milan, Italy). Solvent evaporation was carried out using a rotating evaporator (Heidolph Laborota 400, Heidolph Instruments, Schwabach, Germany) equipped with a vacuum pump (Diaphragm Vacuum Pump DC-4). Lyophilization was performed with a LyoQuest-85^®^ freeze drier (Azbil Telstar Technologies, Barcelona, Spain). C2C12 myoblasts, an immortalized murine cell line, were purchased from ECACC 91031101. Dulbecco’s modified Eagle medium (DMEM), fetal bovine serum (FBS), glutamine, amphotericin B and penicillin-streptomycin were purchased from Gibco, Thermo Fisher Scientific (Waltham, MA, USA). Thiazolyl Blue Tetrazolium Bromide (MTT solution), Phalloidin-Atto 488, Phalloidin-Atto 594 and Alexafluor 488 were obtained from Merck.

### 4.2. Preparation and Characterization of Free Base form of PTM

PTM-B was obtained by dissolving PTM-I in distilled water and adding a 25% *w*/*w* NH_4_OH solution at 4 °C. The obtained precipitate was filtered, washed with a 5% NH_4_OH solution and dried under vacuum conditions overnight. The conversion of PTM-I into PTM-B was confirmed by mass spectrometry analysis using electrospray ionization or by atmospheric pressure chemical ionization, in positive ion mode, on a Micromass ZQ spectrometer (Waters, Milan, Italy), as previously reported [56].

### 4.3. Preparation of Nanoparticles

SC-associated PLGA NPs were prepared by the nanoprecipitation technique [32]. Practically, for each preparation, an aliquot of a methanolic stock solution of SC (1 mg/mL), corresponding to SC 5% or 10% *w*/*w*, was added to 5 mg of PLGA 75:25 dissolved in acetone, until a total volume of 1 mL was reached. The organic solution was then dripped into 5 mL of a 0.2% *w*/*w* PVA solution in MilliQ^®^ water under magnetic stirring. The formation of NPs occurred immediately. After solvent evaporation under reduced pressure, an aqueous NP suspension was obtained. PTM-B-loaded SC-associated NPs were prepared as well, adding an aliquot of a methanolic stock solution of PTM-B (2.5 mg/mL) to the organic solution containing PLGA and SC, for a maximum of 30 µg/mL PTM-B concentration in the final NP suspension. To purify the NPs from unincorporated drugs and SC, PTM-B-loaded SC-associated NPs were extensively dialyzed against MilliQ^®^ water at 4 °C (Spectra/Por^®^ 3500 MWCO dialysis membrane; Spectrum, Houston, TX, USA). Unloaded and untargeted NPs, i.e., without PTM and/or SC, were prepared as well. Fluorescent NPs were prepared by adding 1.4 µg of the fluorescent probe Nile Red/mg of PLGA. The suspensions were then stored at 4 °C until further use.

### 4.4. Characterization of Nanoparticles

The mean hydrodynamic diameter and the PDI of all the NP samples were analyzed by dynamic light scattering (DLS) using a nanosizer (Zetasizer Nano Z, Malvern Inst., Malvern, UK). The selected angle was 173°, and the measurements were carried out at 25 °C after dilution of the particulate suspensions in MilliQ^®^ water. Each measurement was performed in triplicate.

The surface charge of the NPs was evaluated by zeta potential measurements at 25 °C after appropriate dilution in MilliQ^®^ water of the suspensions, using the Smoluchowski equation and the Nanosizer Nano Z. Each reported value is the average of three measurements.

The physical colloidal stability of the NP suspensions in the storage conditions (4 °C) was monitored by evaluating mean diameter, PDI and zeta potential by DLS at different interval times for 4 weeks. Each measurement was carried out in triplicate.

The amount of incorporated PTM-B was determined spectrophotometrically (DU 730 UV-vis spectrophotometer, Beckman Coulter, Brea, CA) at 264 nm using a calibration curve. To this aim, each suspension was lyophilized for 24 h; then, the powder was dissolved in dichloromethane and methanol was added to precipitate PLGA. Then, the suspension was centrifugated (6000 rpm for 15 min) to separate the precipitated polymer. The supernatants were then measured at 264 nm [33]. The concentration of the polymer in the suspensions was based on dry weight analysis. Each sample was analyzed in triplicate. The results were expressed as EE and DL.

### 4.5. Determination of the Associated SC Percentage

The amount of SC associated with the NPs was determined by UV-HPLC on a Shimadzu LC-10ADvp pump equipped with a Shimadzu SPD-10Avp UV–Vis detector set at 215 nm. The analysis was performed on lyophilized NP samples, dissolved in the HPLC mobile phase. Chromatographic separations were performed at room temperature on a reverse phase Kinetex 5 μm C18 100 Å, 150 × 4.6 mm (Phenomenex, Castelmaggiore, Bologna, Italy), equipped with a C18 4.0 × 3.0 mm SecurityGuard cartridge. The volume of injection was 20 μL, and the mobile phase was a mixture of sodium phosphate buffer 50 mM pH 2.7 and methanol (98:2, *v*/*v*) containing sodium 1-heptanesulfonate 2.5 mM, delivered at a flow rate of 0.7 mL/min. Data acquisition and processing were carried out using Autochro 3000 software (Young Lin Instrument, Anyang, South Korea) running on a Windows XP-equipped computer, and the amount of SC was calculated from a calibration curve in the range of 50 to 400 μg/mL.

### 4.6. PTM-B Release from Nanoparticles

To evaluate the PTM-B release from 5% SC-NPs as a function of time, the suspensions were incubated at 37 °C in a 10 mM PBS buffer pH 7.4 in sink conditions. Aliquots (1 mL) were withdrawn at predetermined time intervals (0, 1, 2, 4, 6, 8, 16, and 24 h) and, after purification by dialysis, the drug content was measured as previously described and compared to the initial value.

### 4.7. Cell Culture and Treatment

C2C12 myoblasts were cultured in 75 cm^2^-plastic flasks using DMEM, supplemented with 10% (*v*/*v*) FBS, 1% (w/v) glutamine, 0.5% (*v*/*v*) amphotericin B, 100 units/mL of penicillin-streptomycin and incubated at 37 °C with 5% CO_2_. Cells were trypsinized in 0.05% EDTA in PBS and seeded in flat-bottom 96-well plates (3 × 10^3^ cells/well) for the MTT assay or onto glass coverslips (12-mm diameter) in 24-multiwell plates (8 × 10^3^ cells/well). For the differentiation into myotubes, myoblasts seeded on coverslips were grown at confluence and then a differentiation medium (containing 1% FBS) was added for 7 days. Twenty-four hours after seeding, the cells were treated with 5% SC-NPs for increasing times (see below) and analysis then processed.

### 4.8. Cytotoxicity Assay

The tetrazolium salt MTT (3-(4,5-dimethylthiazol-2-yl)-2,5-diphenyltetrazolium bromide) assay was used to assess the cytotoxicity of 5% SC-NPs on myoblasts. This colorimetric assay is based on the reduction of the MTT to purple formazan crystals by the mitochondrial NAD(P)H-dependent oxidoreductase enzymes: the darker the sample, the greater the number of metabolically active cells. This measurement of metabolic activity therefore represents an indicator of cell viability and cytotoxicity. The cells were treated with different nanocarrier concentrations, selected based on their PTM-B entrapment capacity (from 47.35 to 284.14 μg/mL of PLGA) for 2 h, 24 h and 24 h + 24 h of recovery (i.e., the medium containing the NPs was removed and replaced by a fresh one devoid of NPs). Untreated cells were used as the control. At the end of each incubation period, the medium was replaced by 100 µL of 0.5 mg/mL MTT solution (0.5 mg/mL in medium) and incubated for 4 h at 37 °C in a cell incubator. Then, the MTT solution was removed, and formazan crystals were dissolved in 100 µL of DMSO. The absorbance was measured at 570 nm using a ChroMate 4300 ELISA microplate reader (Awareness Technology Inc., Palm City, FL, USA). Experiments were performed in triplicate. Statistical comparisons between control and experimental conditions were made by the Mann–Whitney pairwise test and significant difference was set at *p* ≤ 0.05.

### 4.9. Fluorescence and Transmission Electron Microscopy Analysis

For microscopy analyses of NP uptake and intracellular distribution, myoblasts and myotubes, adhering to glass coverslips, were incubated for 2 h and 24 h with NPs at the concentration of 94.71 μg/mL.

For CFM, the cells were incubated for 2 h and 24 h with Nile Red-labeled PLGA NPs (5% SC- and untargeted). At the end of each incubation time, cells were fixed with 4% (*v*/*v*) paraformaldehyde in PBS pH 7.4 for 30 min at room temperature. To visualize the intracellular distribution of fluorescent nanocarriers, the cells were washed in PBS, incubated with Phalloidin-Atto 488 diluted 1:20 in PBS, stained for DNA with Hoechst 33,342 (1 μg/mL in PBS), rinsed in PBS, and finally mounted in 1:1 mixture of glycerol:PBS.

For observations, a Leica TCS SP5 AOBS system (Leica Microsystems Srl, Milan, Italy) was used: for fluorescence excitation, a diode laser at 405 nm for Hoechst 33342, an Ar laser at 488 nm for Phalloidin-Atto 488 and Alexafluor 488, and a He/Ne laser at 543 nm for Nile Red and Phalloidin-Atto 594 were employed. Z-stack of 1 µm step-sized images (1024 × 1024 pixel format) were collected.

A morphometric analysis was performed to compare the amount of internalized 5% SC-NPs with untargeted NPs in both myoblasts and myotubes after 24 h incubation. The area of fifty cells per sample was measured by using Image J software (NIH), the number of fluorescent spots occurring in the cytoplasm was counted manually and their surface density (expressed as number/µm^2^) was calculated. Statistical comparisons were made by the Mann–Whitney pairwise test (significant difference at *p* ≤ 0.05).

For TEM, the cells were incubated for 2 and 24 h with the 5% SC-NPs at the same concentration used for CFM (94.71 μg/mL). Then, cells were fixed with 2.5% (*v*/*v*) glutaraldehyde and 2% (*v*/*v*) paraformaldehyde in 0.1 M PBS pH 7.4 at 4 °C for 2 h, post-fixed with 1% OsO_4_ at room temperature for 1 h, dehydrated with acetone and embedded as a monolayer in Epon [57]. Ultrathin sections were observed, unstained or after weak staining with the uranyl acetate replacement stain (Electron Microscopy Sciences, Hatfield, PA, USA) (UAR-EMS). Observations were made in a Philips Morgagni TEM (FEI Company Italia Srl, Milan, Italy), operating at 80 kV and equipped with a Megaview II camera for digital image acquisition.

### 4.10. Immunofluorescence Microscopy

For immunofluorescence microscopy, the cells were fixed with 4% (*w*/*v*) paraformaldehyde in PBS pH 7.4 for 30 min at room temperature. Cells were then permeabilized in PBS containing 0.05% Tween 20 and 0.1% bovine serum albumin (BSA) and incubated with a rabbit polyclonal antibody directed against the OCTN2 receptor (Abcam ab180757), diluted 1:200, for 1 h at room temperature. Cells were washed with PBS and incubated with the secondary antibody (Alexafluor 488), diluted 1:200 in PBS, for 1 h at room temperature. After washing, cells were incubated with Phalloidin-Atto 594, diluted 1:50 in PBS, for 1 h at room temperature. Finally, cells were washed and mounted in 1:1 mixture of glycerol:PBS. Observations were made with a Leica TCS SP5 AOBS system, as above. To quantify OCTN2 receptor signal, the area of thirty cells per sample was measured using Image J software (NIH), and the number of fluorescent spots occurring in the cell was counted manually and their surface density (expressed as number/µm^2^) was calculated. Statistical comparisons were made by the Mann–Whitney pairwise test (significant difference at *p* ≤ 0.05).

## 5. Conclusions

To our knowledge, our study demonstrates for the first time that the association of L-carnitine to PLGA NPs may allow skeletal muscle tropism. In fact, the uptake of L-carnitine-functionalized NPs was markedly increased in terminally differentiated muscle cells, where OCTN2 receptors are highly expressed, compared to myoblasts. Moreover, the developed nanosystem is characterized by a high biocompatibility with skeletal muscle cells, making the SC-NPs potential candidates for delivering therapeutic agents against muscular pathologies. However, the slight cytotoxicity of SC-NPs observed in myoblasts, although being transitory and reversible, deserves deeper study, as do the effects of these NPs on cell metabolism, specifically on mitochondrial activity, in both healthy and myotonic dystrophy type I-affected myotubes. The next steps will also concern the study of the targeting capability of SC-NPs inside the muscle organ, where muscle cells are surrounded by the connective tissue acting as a barrier. To this aim, a fluid dynamic system [58] will be used as an in vitro model to investigate the interactions between NPs and biological barriers [59]. Finally, the activity of PTM-B-loaded targeted NPs will be evaluated on the basis of a murine muscle cell line, transfected with human (CTG)n DNA, as an in vitro model expressing the myotonic dystrophy type I phenotype [29].

## Figures and Tables

**Table 1:** Physicochemical characteristics (mean diameter, PDI and zeta potential) of PLGA nanoparticles (n = 3).

Nanoparticle Composition	Mean Diameter (nm ± S.D.)	PDI	Zeta Potential (mV ± S.D.)
PLGA	94 ± 1	0.170	−39.2 ± 1.8
5% SC-PLGA	82 ± 1	0.198	−23.7 ± 1.1
10% SC-PLGA	73 ± 1	0.184	−29.6 ± 1.0
5% SC-PTM-B-PLGA	98 ± 11	0.399	−18.4 ± 2.4
10% SC-PTM-B-PLGA	128 ± 10	0.222	−28.8 ± 3.3

## Data Availability

Data are contained within the article. Additional data are available from the corresponding author, upon reasonable request.
